# COVID-19-ECMO mit Seltenheitswert: wenn Blutgruppe „0“ zum Problem wird

**DOI:** 10.1007/s00101-023-01325-w

**Published:** 2023-09-01

**Authors:** F. Dietrich, J. M. Wischermann, R. Deitenbeck, U. H. Frey

**Affiliations:** 1 Klinik für Anästhesiologie, operative Intensivmedizin, Schmerz- und Palliativmedizin, Marienhospital Herne, Universitätsklinikum der Ruhr-Universität Bochum, Hölkeskampring 40, 44625 Herne, Deutschland; 2 DRK-Blutspendedienst West, Hagen, Deutschland

## Anamnese

Ein 61 Jahre alter Patient indischer Herkunft entwickelte im Verlauf einer COVID-19-Pneumonie ein ARDS (acute respiratory distress syndrome) und wurde zur Initiierung einer vv-ECMO-Therapie (venovenöse extracorporeal membrane oxygenation) evaluiert. Bei vorliegender katecholaminpflichtiger Kreislaufinsuffizienz, einem Lactat von 2,1 mmol/l, einem pH-Wert von 7,48, einer Krankenhausverweildauer > 7 Tage und Thrombozyten von 154 (• 1000 µl^−1^) erreichte der Patient in der Summe 8 Punkte im PRESET Score und somit ein erhöhtes Mortalitätsrisiko [5, 8]. Der „PREdiction of Survival on ECMO Therapy-Score“ (PRESET Score) ist ein Mortalitätsvorhersagemodell, das bei einer kostenintensiven und mit potenziellen Komplikationen verbundenen ECMO-Therapie vor deren Etablierung zur Entscheidungsunterstützung verwendet werden kann, um das Mortalitätsrisiko des Patienten einschätzen zu können [5].

## Befund

Bei Vorliegen einer Anämie (Hämoglobin [Hb] von 8,9 g/dl) wurden eine serologische Verträglichkeitsprobe zwecks Bereitstellung entsprechender Erythrozytenkonzentrate (EK) sowie eine labortechnische AB0-Blutgruppenmerkmal-Bestimmung angefordert. Der Bedside-Test, als bettseitiger AB0-Identitätstest, ergab die Blutgruppe 0; ungekreuzte EK waren vorrätig. Der klinische Zustand verschlechterte sich akut, sodass unter Verzicht des Ergebnisses der serologischen Verträglichkeitsprobe die sofortige sonographisch gesteuerte komplikationslose bifemorale venöse Punktion und Insertion der ECMO-Kanülen erfolgten. Nach der Kanülierung ergab die arterielle Blutgasanalyse einen Hb dilutionsbedingt von 7,2 g/dl; das Labor übermittelte die Blutgruppe 0h.

## Blutgruppe 0_h_ (Bombay)

Die *Blutgruppe 0h (Bombay)*, auch *Bombay-*Phänotyp genannt, findet sich mit einer Prävalenz von 1:300.000 weltweit bei ca. 26.000 Menschen und stellt eine sehr seltene Variante im AB0-System dar. In manchen Bevölkerungsteilen Indiens tritt 0h gehäuft auf. Als Grundbaustein ist das Antigen H bei allen Oberflächenantigenen im AB0-Blutgruppensystem vorhanden. Bei der Blutgruppe 0h entsteht durch einen Gendefekt kein Antigen H (Blutgruppe 0), sondern lediglich die Vorläufersubstanz/Grundsubstanz h. Die Blutgruppe im Bedside-Test präsentiert sich als dem Phänotyp 0 zugehörig [8]. Aufgrund eines Antikörpers gegen das Merkmal H können 0h-Patienten nur mit 0h-Blut, nicht jedoch mit Blut der Blutgruppe 0 versorgt werden [1]. Eine Transfusion einer Konserve Blutgruppe 0 bei einem 0h-Empfänger würde eine hämolytische Transfusionsreaktion vom Soforttyp nach sich ziehen, identisch zu einer Fehltransfusion im AB0-Blutgruppensystem.

## Klinischer Verlauf und Therapie

Eine Versorgung mit passenden EK war nur mit erheblicher Zeitverzögerung, vermehrten Kosten und Aufwand sowie nur in reduzierter Menge möglich. Da es sich im vorliegenden Fall um eine rezessiv vererbbare und familiär gehäuft vorkommende Mutation im *FUT1*-Gen (Genotyp h/h) handelte, wurde zunächst die intrafamiliäre Suche nach geeigneten Spendern veranlasst. Die Austestung der beiden Söhne des Patienten ergab jedoch die Blutgruppe A. Eine weitere Akquirierung von potenziellen Spendern wurde durch die Suche im Register „Seltene Blutspender“ der deutschsprachigen wissenschaftlichen Fachgesellschaft angestoßen, führte jedoch ebenfalls nicht zu einem zeitnahen positiven Ergebnis.

**Figure 1:**
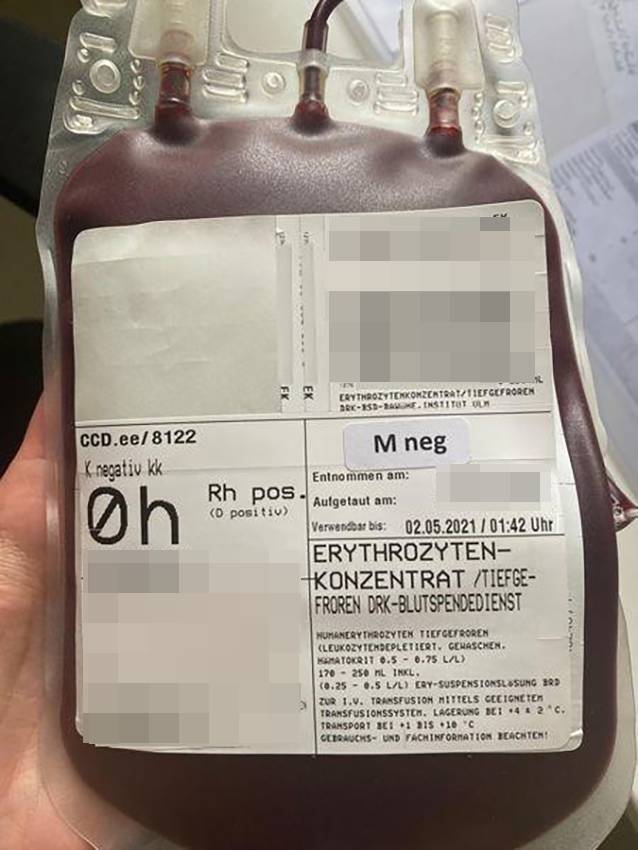

Kompatible kryokonservierte EK werden in Deutschland in den Blutspendediensten Hagen und Ulm vorgehalten. Nach ersten serologischen sowie molekulargenetischen Untersuchungen konnten 2 geeignete EK (Abb. 1) durch den Blutspendedienst in Hagen aus der Kryoblutbank in Ulm innerhalb von 24 h organisiert und transfundiert werden.

**Figure 2:**
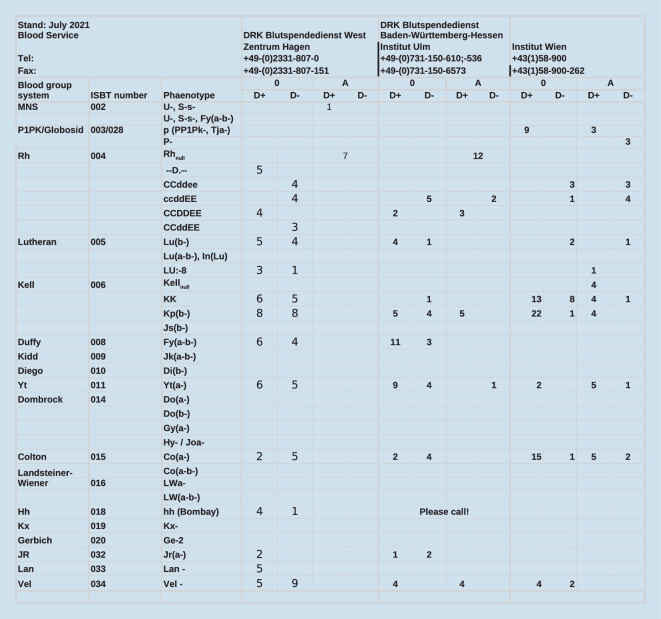

Eine Bestandsübersicht über kryokonservierte EK für seltene Blutgruppen ist auf der Seite der interregionalen Blutspende des Schweizerischen Roten Kreuzes zu finden (Abb. 2).

## Ethisches Gewicht/ethische Fallbesprechung

Aufgrund der höchst eingeschränkten Reserve an kompatiblen EK, der schlechten allgemeinen Prognose und des prognostisch erhöhten Transfusionsbedarfs wurde bereits 2 Tage nach der erfolgten ECMO-Therapie eine interdisziplinäre ethische Fallbesprechung einberufen: Neben den medizinethischen Prinzipien Respekt vor der Autonomie des Patienten, Prinzip des Nichtschadens sowie Prinzip des Wohltuns hatte das Prinzip der Gerechtigkeit, insbesondere der Verteilungsgerechtigkeit, in dieser Fallbesprechung besonderes Gewicht erlangt.

Diskutiert wurde die zentrale Frage, ob zugunsten eines Patienten, der eine reduzierte Überlebenswahrscheinlichkeit hat [3], die limitierten europaweiten Reserven zulasten anderer potenzieller Patienten ausgeschöpft werden dürfen und die damit möglicherweise einhergehende Unterversorgung anderer Patienten gerechtfertigt sei.

Im Konsens wurde entschieden, die europaweiten Reserven unangetastet zu lassen, die bereits etablierte Therapie und alle möglichen supportiven Maßnahmen fortzuführen oder einzuleiten.

## Weiterer Verlauf

**Figure 3:**
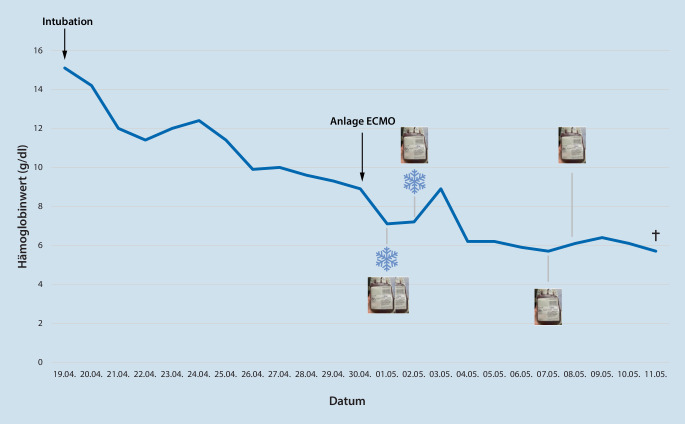

Im weiteren Verlauf kam es zunächst zu einer Besserung des Gasaustausches bei zunehmend sinkender Hb-Konzentration. Durch einen Aufruf in den sozialen Medien seitens der Familie und Suche nach geeigneten Spendern konnten 5 bzw. 6 Tage nach der letzten erfolgten Transfusion zwei weitere EK aus frischen Spenden (Baden-Württemberg und Nordrhein-Westfalen) akquiriert und bei einem Hb von 5,7 g/dl bzw. 6,1 g/dl erfolgreich transfundiert werden (Abb. 3).

Trotz eskalierter Therapiemaßnahmen verstarb der Patient 12 Tage nach Initiierung der ECMO-Therapie im septischen Schock auf dem Boden eines Multiorganversagens bei einem Hb-Wert von 5,7 g/dl.

## Diskussion

Wir berichten hier über einen Patienten mit der sehr seltenen Blutgruppe 0h (Bombay), dessen Behandlung v. a. durch die Organisation von seltenen kryokonservierten EK und die Suche nach geeigneten Spendern geprägt war, da bei Patienten unter vv-ECMO ein durchschnittlicher EK-Bedarf von 1500 ml über 10 Tage ECMO-Therapie postuliert werden kann [2]. Ein weiteres organisatorisches Problem bestand in den kryokonservierten EK selbst. Diese werden nur in speziellen Zentren gelagert und für die Transfusion aufbereitet. Nach dem Auftauen einer solchen Konserve muss diese auch in 24 h transfundiert werden. Eine hausinterne Bereitstellung und Lagerung sind damit nicht möglich. Hinzu kommen die überregionale Auswirkung auf die Blutversorgung mittels EK und die reduzierte Anzahl der Blutspenden aufgrund der SARS-CoV-2-Pandemie [6].

Die Gabe von ungekreuzten EK mit vorliegendem Bedside-Test in einer akuten Blutungssituation, wie sie bei einer ECMO-Kanülierung auftreten kann, hätte im vorliegenden Fall möglicherweise fatale Konsequenzen gehabt [7].

Weiterhin hat der ethische Aspekt eine zentrale Rolle eingenommen. So musste im Sinne des Prinzips Gerechtigkeit eruiert werden, wie mit einer europaweit knappen Ressource umgegangen wird, die anderen Patienten potenziell eine Überlebenschance sichert. Von besonderer Bedeutung bleibt letztlich die philosophische Frage, ob die Entscheidung zur ECMO-Therapie mit einem *A-priori*-Wissen der Blutgruppe Bombay ebenso getroffen worden wäre.

Wird der Verdacht auf das Vorliegen einer seltenen Blutgruppe geäußert, werden bestätigende Untersuchungen in einem größeren Zentrum durchgeführt. Bestätigt sich der Verdacht, kann im Register der Deutschen Gesellschaft für Transfusionsmedizin und Immunhämatologie (DGTI) nach geeigneten Spendern gesucht werden. Die öffentlich zugängliche Liste der deutschsprachigen Kryobanken listet den aktuellen Bestand an kryokonservierten EK im deutschsprachigen Raum auf. Eine europaweite Anfrage für kryokonservierte EK kann durch einen Transfusionsmediziner in einem entsprechenden Zentrum gestartet werden.

## Ausblick in die Zukunft – Wissenschaft als Antwort?

Hawksworth et al. [4] nutzten in ihrer Arbeit die 2020 mit dem Chemienobelpreis prämierte CRISPR/Cas-Methode, um gezielte Knock-outs in Blutgruppengenen innerhalb einer immortalisierten Erythroblastenreihe zu erzeugen. So entstanden für einzelne Blutgruppenantigene translatierende funktionslose Enzyme mit dem Resultat von *in vitro* fehlender Agglutination bei Antikörperkontakt. Ob diese Methode den Weg in die Klinik findet, bleibt abzuwarten, dennoch scheint diese innovative Technik eine potenzielle Hoffnung für viele Patienten zu bieten und eine Antwort auf fehlende Ressourcen der Blutbanken zu geben.

## Fazit

Die meisten Kliniken halten für Notfallsituationen EK der Blutgruppe 0 vor. Diese sind in den meisten Fällen verträglich. Das Beispiel der Blutgruppe 0h zeigt jedoch, dass auch bei augenscheinlicher Blutgruppe 0 eine seltene Blutgruppe oder seltene Antikörper vorliegen können, die bei Fehltransfusion dramatische Folgen für den Patienten haben. Transfusionen von ungekreuzten EK bei ausstehender serologischer Blutgruppenbestimmung sollten Notfallsituationen mit vitaler Indikation vorbehalten bleiben. Solche Notfallsituationen ausgenommen, ist in den meisten anderen klinischen Situationen in der Regel ein Abwarten der labortechnischen Blutgruppenbestimmung vertretbar. Einen Hinweis auf 0h ist eine stark positive Reaktion mit der 0‑Zelle in der Serumgegenprobe der AB0-Blutgruppenbestimmung im Labor. Liegt eine seltene Blutgruppe vor, sollten umgehend alle Maßnahmen des Patient Blood Management umfassend umgesetzt sowie zeitnah Kontakt zu einem Blutspendedienst aufgenommen werden, da für die Bereitstellung kompatibler EK besondere personelle Ressourcen und Logistik notwendig sind.

## References

[1] Davey RJ, Tourault MA, Holland PV (1978). The clinical significance of anti-H in an individual with the Oh (Bombay) phenotype. Transfusion.

[2] Davies A, Jones D, Bailey M (2009). Extracorporeal membrane oxygenation for 2009 influenza A(H1N1) acute respiratory distress syndrome. JAMA.

[3] Friedrichson B, Kloka JA, Neef V, Mutlak H, Old O, Zacharowski K, Piekarski F. Extracorporeal membrane oxygenation in coronavirus disease 2019: A nationwide cohort analysis of 4279 runs from Germany. Eur J Anaesthesiol. 2022 May 1;39(5):445-451. 10.1097/EJA.0000000000001670 10.1097/EJA.0000000000001670 35180152

[4] Hawksworth J, Satchwell TJ, Meinders M (2018). Enhancement of red blood cell transfusion compatibility using CRISPR-mediated erythroblast gene editing. EMBO Mol Med.

[5] Hilder M, Herbstreit F, Adamzik M (2017). Comparison of mortality prediction models in acute respiratory distress syndrome undergoing extracorporeal membrane oxygenation and development of a novel prediction score: the PREdiction of Survival on ECMO Therapy-Score (PRESET-Score). Crit Care.

[6] Ngo A, Masel D, Cahill C, Blumberg N, Refaai MA (2020). Blood banking and transfusion medicine challenges during the COVID-19 pandemic. Clin Lab Med.

[7] Shahshahani HJ, Vahidfar MR, Khodaie SA (2013). Transfusion reaction in a case with the rare Bombay blood group. Asian J Transfus Sci.

[8] Tabatabai A, Ghneim MH, Kaczorowski DJ (2021). Mortality risk assessment in Covid-19 veno-venous extracorporeal membrane oxygenation. Ann Thorac Surg.

[9] https://www.iblutspende.ch/fileadmin/user_upload/itransfusion/PDFs/RareDonors/Kryoblutbank2021.pdf. Zugegriffen: 07. Feb. 2022

[10] https://www.iblutspende.ch/rare-donors/seltene-spender.html. Zugegriffen: 07. Feb. 2022

